# Single-cell profiling unveils key regulators of skeletal stem cells in chicken and human embryonic limb development

**DOI:** 10.1371/journal.pone.0346514

**Published:** 2026-04-28

**Authors:** Ruohan Zhao, Zonghui Jian, Fangxiao Yang, Kun Wang, Lixian Liu, Zhiqiang Xu, Junjing Jia, Tengfei Dou, Xiaoming He

**Affiliations:** 1 College of Animal Science and Veterinary Medicine, Yunnan Vocational and Technical College of Agriculture, Kunming, Yunnan, China; 2 College of Animal Science and Technology, Yunnan Agricultural University, Kunming, Yunnan, China; 3 Institute of Science and Technology, Chuxiong Normal University, Chuxiong, Yunnan, China; 4 College of Food Science and Technology, Yunnan Agricultural University, Kunming, Yunnan, China; Kyungpook National University School of Medicine, KOREA, REPUBLIC OF

## Abstract

Skeletal stem cells (SSCs) are well characterized in humans and mice, providing valuable insights into bone development and regeneration. However, their identification in poultry, particularly in chicken embryos, remains limited. In the present study, we used single-cell transcriptomic profiling to compare the cellular composition and functionality of human and chicken embryonic limb buds. Our cross-species analysis revealed high conservation of key cell types, such as mesenchymal and osteochondral progenitors, alongside notable heterogeneity in individual gene expression profiles. We identified the conserved and species-specific gene expression patterns in chicken SSCs. Differential expression analysis combined with Gene Ontology (GO), Kyoto Encyclopedia of Genes and Genomes (KEGG) pathways, and protein-protein interaction (PPI) networks suggested that *COL5A2*, *COL1A2*, *PRRX1*, and *TGFβ2* may serve as key regulators of chicken SSCs. Immunofluorescence assays confirmed the predominant localization of these genes in the periosteal region, with minor expression in the primary ossification centers. This study enhances our understanding of cellular diversity in chicken embryonic limb buds and highlights conserved and divergent mechanisms across species, offering insights for future SSC research and animal model selection in skeletal biology.

## Introduction

The skeleton is a dynamic organ composed of bone, cartilage, adipose, fibrous, neural, vascular, and hematopoietic tissues with notable regenerative capacity [1]. Musculoskeletal diseases such as osteoarthritis, fractures, ankylosing spondylitis, osteoporosis, and muscle atrophy are the major causes of disability and significantly affect mobility and quality of life [2–4]. Current treatments are limited by a poor understanding of stem cell regulation in the skeletal system. Stem cells are vital for the generation and maintenance of skeletal tissue because of their ability to proliferate and differentiate [5,6]. Mouse skeletal stem cells (mSSCs) have been identified via lineage tracing [7], whereas human skeletal stem cells (hSSCs) have been discovered through single-cell transcriptomic sequencing (scRNA-seq), revealing key markers, such as *CADM1*, *CD200*, *PDGFRA*, and *CXCL12* [8,9]. However, studies on hSSCs are limited by ethical and practical constraints that restrict systematic investigation of their differentiation trajectories and functional characteristics.

As bipedal animals, chickens offer biomechanical and anatomical parallels to humans, making them useful models for studying skeletal development [10,11]. Their lower limbs experience similar mechanical stresses, and comparative genomics indicate high sequence conservation between chickens and humans, particularly in coding regions and introns [12,13]. Controlled incubation allows precise access to specific embryonic stages, enabling experimental manipulation and longitudinal studies of limb development [14,15]. Despite the recognized value of chicken models, systematic cross-species comparisons of skeletal stem cell differentiation between humans and chickens are lacking, particularly at single-cell resolution [16].

Feregrino et al. [17] performed scRNA-seq on chicken hind limb tissues and identified mesenchymal populations without comprehensive classification. In this study, we integrated scRNA-seq data from chicken hind limbs with data from human embryonic limb buds and long bones to map skeletal stem cells (SSCs) and assess the conserved differentiation programs. This study fills a critical gap by providing a direct cross-species analysis of skeletal stem cell populations, offering insights into conserved and divergent mechanisms in limb development, and supporting the utility of chicken embryos as models for human skeletal biology.

## Materials and methods

### Materials and sample collection

Chicken embryos were obtained from fertilized eggs incubated under standard conditions and staged according to the Hamburger–Hamilton (HH) developmental system (Hamburger and Hamilton, 1951). HH29 embryos correspond to approximately 6–6.5 days of incubation. This study was carried out in strict accordance with the recommendations of the *Guidelines for the Euthanasia of Experimental Animals* issued by the Chinese Association for Laboratory Animal Sciences. The protocol was approved by the Animal Care and Use Committee of Yunnan Agricultural University (Approval No. YAU202103047).

At HH29, embryos were anesthetized with sodium pentobarbital prior to tissue collection. All surgical procedures were performed under sodium pentobarbital anesthesia, and all efforts were made to minimize suffering. Limb bud tissues were collected immediately following administration of the anesthetic, and loss of vital signs and death occurred within approximately 1 minute after anesthesia administration. A total of six embryos were used in this study (n = 6). Throughout the procedure, care was taken to minimize handling time and avoid unnecessary manipulation of the embryos. The collected limb bud tissues were washed with cold phosphate-buffered saline (PBS) and fixed in 4% paraformaldehyde for 48 h prior to subsequent analyses.

### Single-cell transcriptome data preparation

The scRNA-seq data for human embryonic bone were obtained from the Gene Expression Omnibus (GEO) under accession number GSE143753. This dataset included two samples: one from a limb bud (GSM4274188) and the other from a long bone (GSM4274191) [9]. Similarly, scRNA-seq data for embryonic chicken hind limbs at three distinct developmental stages, Hamburger and Hamilton stages 25 (HH25) (GSM3738693), HH29 (GSM3738694), and HH31 (GSM3738695), were downloaded from the GEO database [17,18].

### Quality control and preprocessing of the data (cell clustering)

In this study, we used Seurat (v4.3.0) in R to create the analysis objects and filter low-quality data [19]. The preprocessing steps included calculating gene counts, cell counts, and mitochondrial content percentages. The dataset was normalized using the *NormalizeData* function, and variable genes were identified using the *FindVariableFeatures* function.

Prior to cross-species integration, gene annotations between the human and chicken datasets were carefully examined by cross-checking gene symbols and sequence information to ensure correct correspondence between genes from the two species. The two expression matrices were subsequently aligned using the genes shared between the datasets. To integrate data across species, we applied Canonical Correlation Analysis (CCA) to correct for batch effects [20]. Anchors were identified using *FindIntegrationAnchors*, and the datasets were merged with *IntegrateData*. Following integration, principal component analysis (PCA) was performed using *RunPCA*, with 30 principal component (npcs) sets and 17 components selected for further analysis. The cells were clustered using *FindClusters* with a resolution parameter of 0.5. t-Distributed stochastic neighbor embedding (t-SNE) was used to visualize relationships among cell clusters. Marker genes were selected based on a log fold change (logFC) ≥ 0.5 and adjusted p-value (adjPval) < 0.05 between clusters. This threshold was selected to account for the relatively subtle transcriptional differences among closely related cell populations in early embryonic limb tissues, while retaining sufficient genes for downstream functional enrichment and comparative analyses [21].

### Differentially expressed gene (DEG) and functional enrichment analyses

All the DEGs for each cell type were identified using the *FindAllMarkers* function in Seurat. Gene Ontology (GO) annotation and Kyoto Encyclopedia of Genes and Genomes (KEGG) pathway enrichment analyses were conducted using the Database for Annotation, Visualization, and Integrated Discovery (DAVID) tools (https://david.ncifcrf.gov/). The statistical significance of the enriched terms was evaluated using Benjamini–Hochberg false discovery rate (FDR) and adjusted p-value (adj. P) < 0.05, which was considered significant and consistent with standard practice for multiple testing correction [22].

### Trajectory and cell–cell communication analysis

The cell differentiation trajectories were inferred using Monocle 3, which models the asynchronous progression of individual cells along a differentiation trajectory within an unsupervised framework [23,24]. To analyze cell–cell communication, we used CellChat (v1.6.1), which infers intercellular signaling networks from a curated ligand–receptor interaction database (CellChatDB) that encompasses secreted signaling, extracellular matrix–receptor interactions, and cell–cell contact–dependent pathways [25]. The CellChatDB used in this study was accessed in March 2024.

### Protein-protein interaction (PPI) network construction and hub gene identification

The Search Tool for the Retrieval of Interacting Genes (STRING) database (http://string-db.org/) was used to analyze PPI networks, which were visualized using Cytoscape software (www.cytoscape.org/). Interactions with confidence ≥0.4 were retained, including experimentally validated, curated database, and high-confidence predicted interactions. CytoHubba (v3.9.1) was used to calculate protein node connectivity using the degree method, as it is simple and effective for identifying hub genes [26].

### Immunofluorescent staining

The previously collected and fixed chicken embryonic limb bud tissues were processed for paraffin embedding and sectioning. The paraffin-embedded sections were dewaxed using an eco-friendly solution (G1128, Servicebio, Wuhan, China), dehydrated using a graded ethanol series, and subjected to antigen retrieval. Sections were washed with PBS, air-dried, and circled using a hydrophobic pen. A blocking solution of 3% bovine serum albumin (BSA) was applied for 30 min at room temperature. The primary antibody was incubated overnight at 4℃, followed by incubation with the secondary antibody at room temperature for 50 min. After additional PBS washes, the cell nuclei were stained with DAPI (G1012, Servicebio, Wuhan, China), and a self-quenching mounting medium (G1221, Servicebio, Wuhan, China) was applied. Images were captured using a Nikon Eclipse C1 fluorescence microscope, and analyses were performed using the CaseViewer software. The following antibodies were used: anti-COL5A2 antibody (GB111012, 1:500, Servicebio, Wuhan, China), anti-COL1A2 antibody (AF7001, 1:200, Affinity Biosciences, Jiangsu, China), anti-PRRX1 antibody (DF4274, 1:200, Affinity Biosciences, Jiangsu, China), anti-TGFβ2 antibody (AF0260, 1:100, Affinity Biosciences, Jiangsu, China), and Cy3-conjugated goat anti-rabbit IgG (GB21303, 1:300, Servicebio, Wuhan, China).

## Results

### Generation of cross-species single-cell transcriptomic atlases of embryonic limb bud

**Fig 1:**
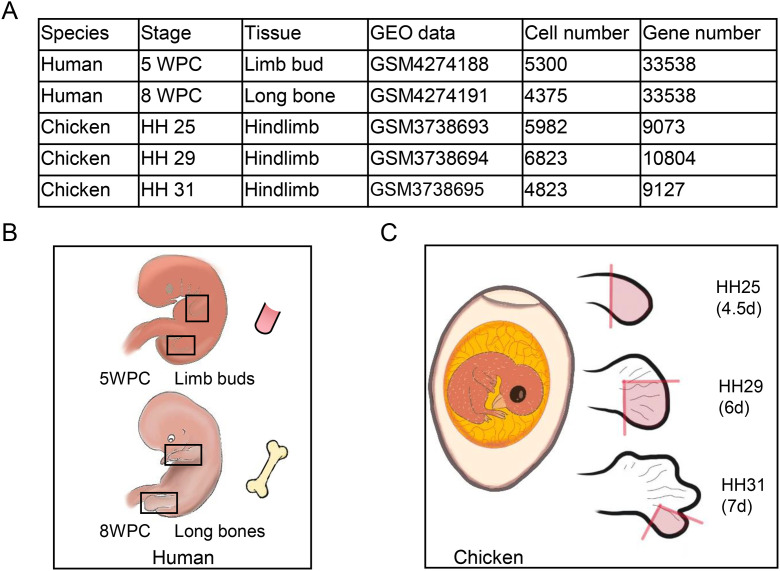
Sample information and sampling strategy. **A.** Table summary of human embryonic limb bud and chicken hindlimb samples with detailed scRNA-seq information. **B.** Human embryonic cells from 5 weeks post-conception (WPC) limb buds and 8 WPC long bones were sampled. **C.** Chicken embryonic cells from different stages of hind limb development were sampled.

The human embryonic skeleton included the limb bud at 5 weeks post-conception (WPC) (HBone_5) and long bones at 8 WPC (HBone_8) (Fig 1B). In contrast, the embryonic chicken hindlimb encompasses the entire distal limb region, with sampling at HH25 (4.5–5.0 days, CBone_25), HH29 (6.0–6.5 days, CBone_29), and HH31 (7.0–7.5 days, CBone_31), all critical for bone formation (Fig 1C). The human limb bud at 5 WPC contained approximately 5,300 cells, whereas the long bone at 8 WPC contained 4,375 cells. The chicken embryonic stages comprised 5,982, 6,823, and 4,823 cells (Fig 1A).

**Fig 2:**
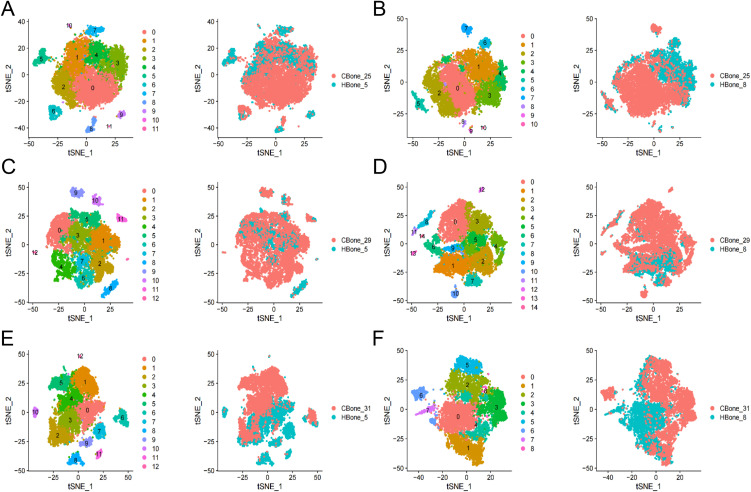
Cluster analysis of cell types of human embryonic limb buds and embryonic chicken hindlimbs. **A.** Integration analysis of human embryonic limb bud (5 WPC) and chicken hindlimb (HH25). **B.** Integration analysis of human embryonic long bone (8 WPC) and chicken hindlimb (HH25). **C.** Integration analysis of human embryonic limb bud (5 WPC) and chicken hindlimb (HH29). **D.** Integration analysis of human embryonic long bone (8 WPC) and chicken hindlimb (HH29). **E.** Integration analysis of human embryonic limb bud (5 WPC) and chicken hindlimb (HH31). **F.** Integration analysis of human embryonic long bone. The degree of overlap reflects transcriptional similarity after CCA-based integration and should not be interpreted as a precise temporal alignment between human and chicken developmental stages. All figures were generated by the authors using original data in R.

Using CCA to normalize variance and adjust for batch effects, we integrated human embryonic limb bud stages with chicken hindlimb data (Fig 2). This integration was performed to evaluate transcriptional similarity and conserved cell-type composition across species rather than to establish exact chronological equivalence between developmental stages.

To quantitatively assess cross-species integration, we further calculated the proportion of human and chicken cells within each integrated cluster across developmental stages (HH25, HH29, and HH31) (S1 Fig). This analysis showed that clusters corresponding to the HH29 stage exhibited the highest degree of cross-species cell mixing, whereas clusters derived from HH25 and HH31 showed stronger species bias. Consistent with these quantitative results, the integrated embedding revealed a substantial overlap between human limb buds at both 5 and 8 WPC and HH29 chicken hind limbs (Fig 2C, 2D). In contrast, the overlap between HH25 and HH31 chicken hind limbs and human embryonic limb buds was less pronounced (Fig 2A–2F).

### Cluster analysis of cell types in human and chicken embryonic limb buds

**Fig 3:**
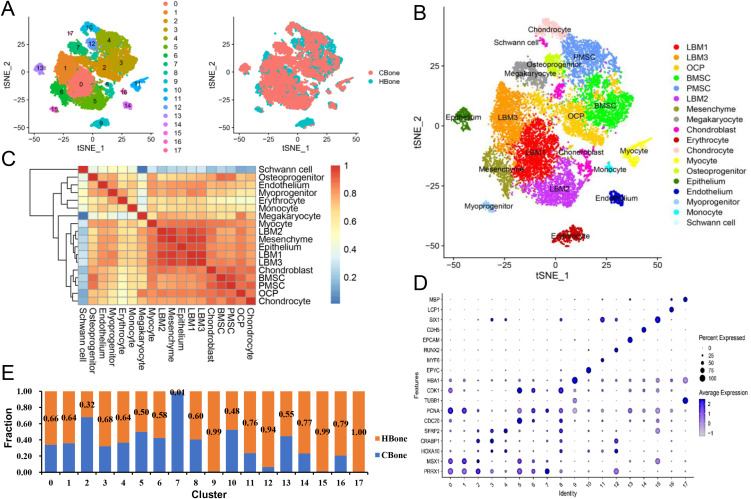
Integration analysis of human embryonic limb buds (5 WPC and 8 WPC) and chicken hindlimb (HH29). **A.** Integration analysis of human embryonic limb buds (5 WPC and 8 WPC) and chicken hindlimb (HH29). **B.** T-Distributed Stochastic Neighbor Embedding (t-SNE) plots showing different cell types from 16498 limb bud bone cells from humans and chickens. **C.** Pearson correlation analysis showing the relationship among the 18 subsets. Hierarchical clustering according to Pearson correlation distinguished skeletogenic (clusters 1-6, 8, 10, and 13) and non-skeletogenic subsets (clusters 7, 9, 11, 12, and 14-17). **D.** Dot plots showing the expression of curated feature genes in 18 subsets, colored by relative gene expression. Each dot represents a gene, and its size indicates the percentage of cells expressing that gene. Cluster numbers on the x-axis correspond to the annotated cell types shown in Fig 3B. **E.** Proportion of cells from human limb buds and chicken limb buds. All figures were generated by the authors using original data in R.

Unsupervised clustering of HH29 chicken limb data integrated with human embryonic limb bud data (5 WPC and 8 WPC) revealed a significant overlap in cell types between the two species (Fig 3A). Cell type annotation utilized reference databases, the CellMarker website, and existing literature (Fig 3B). Overall, most cell types were conserved across species, whereas megakaryocytes, erythrocytes, myoblasts, and Schwann cells showed species-specific distributions. The relative proportions of human and chicken cells within each integrated cluster reflected differences in cellular composition rather than a normalized 1:1 species ratio (Fig 3B, 3E).

The numerical cluster IDs (0–17) shown in Fig 3A correspond exactly to the annotated cell types shown in Fig 3B. Clusters 0, 1, and 5 represented mesenchymal progenitor cells (LBM1–3), showing differential expression of marker genes such as *PRRX1*, *MSX1*, and *PITX1*. Cluster 2 was identified as osteochondral progenitors (OCPs) based on high *HOXA10* and *CRABP1* expression, whereas cluster 3 was characterized as bone marrow stromal cells (BMSCs) due to elevated *CRABP1* and *CXCL12* levels. Cluster 4, marked by *OSR2*, *SFRP2*, and *GAS2*, was defined as perichondrial mesenchymal stromal cells (PMSCs).

Other clusters included *PCNA*^+^ mesenchymal cells (cluster 6), *CDK1*^+^ chondroblasts (cluster 8), *EPYC*^+^ chondrocytes (cluster 10), *MYF6*^+^ myocytes (cluster 11), *EPCAM*^+^ *WNT6*^+^ epithelium (cluster 13), *CDH5*^+^ *RAMP2*^+^ endothelium (cluster 14), and *PTPRC*^+^ *LCP1*^+^ monocytes (cluster 16) (Fig 3B, 3D, 3E). Clusters of *HBA1*^+^ *GYPC*^+^ erythrocytes (cluster 9), *RUNX2*^+^ osteoprogenitors (cluster 12), *SIX1*^+^ myoprogenitors (cluster 15), and *MBP*^+^ Schwann cells (cluster 17) were observed in human limb buds. Conversely, cluster 7, consisting of *PLEK*^+^ *TUBB1*^+^ megakaryocytes, was uniquely detected in the chicken limbs (Fig 3B, 3D, 3E). Pearson’s correlation analysis further distinguished osteogenic from non-osteogenic subpopulations (Fig 3C).

### Conserved cell types and differentiation across species

Limb buds in both the upper and lower extremities originate from the lateral mesoderm and differentiate in a proximal-to-distal sequence. Proximal differentiation occurs earlier, with critical limb formation in humans occurring between 4 and 8 WPC. Disruptions during this period can cause malformations, emphasizing the importance of studying limb bud development, particularly in comparative species analyses.

**Fig 4:**
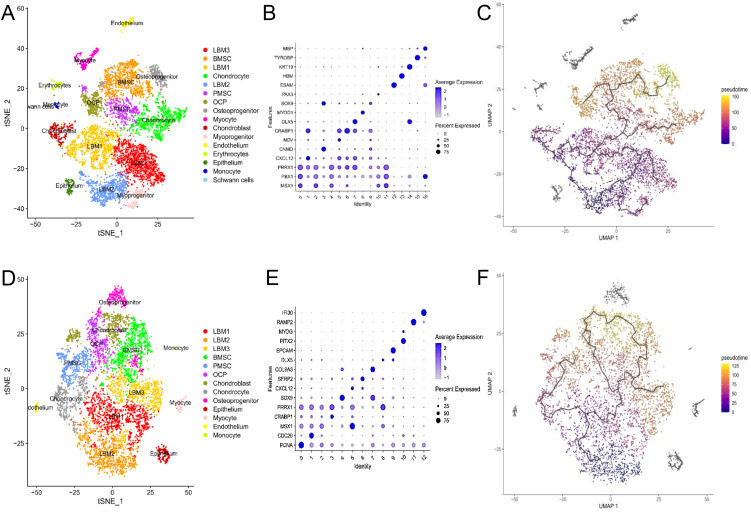
Cell subset clustering and pseudotime analysis of human and chicken limb buds. A. t-SNE visualization of 16 subsets in human embryonic limb buds (5 WPC) and long bones (8 WPC). **B.** Dot plots showing the expression of feature genes for each subset of human limb buds. **C.** Developmental trajectory of 16 subsets inferred by monocle3 and visualized on the uniform manifold approximation and projection (UMAP). D. t-SNE visualization of 13 subsets in chicken embryonic hindlimb (HH29). **E.** Dot plots showing the expression of feature genes for each subset of chicken hindlimb. **F.** Developmental trajectory of 13 subsets inferred by monocle3 and visualized on the UMAP projection. All figures were generated by the authors using original data in R.

To explore the conservation of limb bud development, 16 subpopulations were identified in human limb buds (5 and 8 WPC) based on key marker genes, including LBM1 (*PRRX1*), LBM2 (*MSX1*), LBM3 (*PBX1*), BMSCs (*CXCL12*, *PDGFRA*), PMSCs (*NOV*), OCPs (*CRABP1*), chondroblasts (*SOX9*), chondrocytes (*CNMD*, *COL11A2*), osteoprogenitors (*RUNX2*), epitheliums (*EPCAM*), myoprogenitors (*PAX3*, *MET*), myocytes (*MYOG*), endotheliums (*ESAM*, *CDH5*), erythrocytes (*HBM*, *GYPA*), monocytes (*TYROBP*), and Schwann cells (*MBP*, *CD9*) (Fig 4D, 4E). Similar expression patterns were observed in chicken limb buds, although myoprogenitors, erythrocytes, and Schwann cells were absent, likely because of sampling differences (Fig 4A, 4B, 4C). Pseudotime trajectory analysis revealed developmental relationships, with LBM2 at the origin, followed by LBM1 and LBM3, which further differentiated into PMSCs, BMSCs, and OCPs (Fig 4C, 4F).

### Functional enrichment analysis of mesenchymal progenitor cells

Using marker gene expression profiles and functional enrichment analysis via GO, distinct cell populations were mapped to specific tissue types, enabling a detailed exploration of gene functions in limb development. In the enrichment plots, the y-axis represents various GO terms, whereas the x-axis indicates the fold change in gene expression; larger fold changes reflect greater enrichment, and higher -log10(p-values) indicate increased statistical significance.

**Fig 5:**
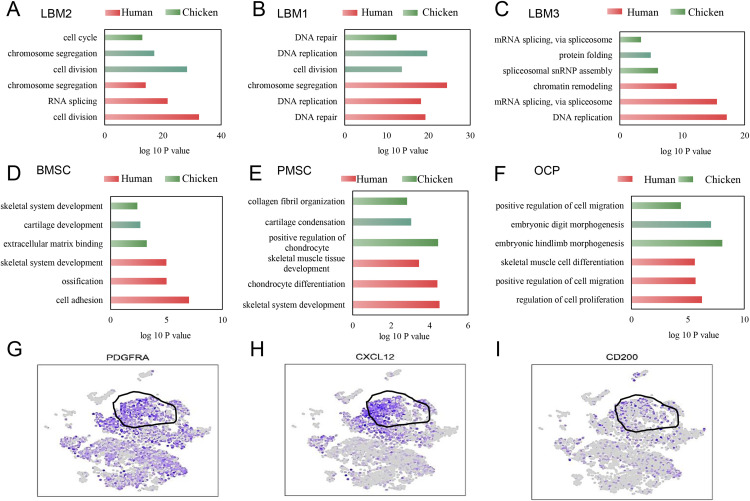
Gene Ontology (GO) functional enrichment analysis of mesenchymal stem cells and gene expression profiling of human bone marrow stromal cells (BMSCs). **A.** GO analysis of mesenchymal progenitor cells (LBM2). **B.** GO analysis of LBM1 cells. **C.** GO analysis of LBM3 cells. **D.** GO analysis of BMSC cells. **E.** GO analysis of perichondrial mesenchymal stromal cells (PMSCs). **F.** GO analysis of osteo-chondrogenic progenitors (OCPs). G. t-SNE plot of PDGFRA gene expression levels. H. t-SNE plot of CXCL12 gene expression levels. I. t-SNE plot of CD200 gene expression levels. All figures were generated by the authors using original data in R.

In limb bud LBM2 cells, DEGs were enriched in pathways related to cell division and cell cycle (Fig 5A). LBM1 DEGs were linked to DNA repair and replication (Fig 5B), whereas LBM3 DEGs were involved in mRNA splicing and chromatin remodeling (Fig 5C). Notably, BMSCs from both species shared enrichment in ossification and skeletal system development pathways (Fig 5D), whereas PMSCs were enriched in chondrocyte differentiation and skeletal muscle development pathways (Fig 5E), and OCPs in hindlimb progenitor differentiation pathways (Fig 5F). These results highlight the conserved functional programs between the human and chicken mesenchymal populations. BMSCs showed moderately higher expression of *PDGFRA*, *CXCL12*, and *CD200* than other mesenchymal subsets, consistent with their stromal-associated enrichment (Fig 5G–5I).

### Expression pattern of chicken BMSCs

The integrated analysis identified chicken BMSC subpopulations as potential SSCs with distinct characteristics. CellChat analysis of receptor-ligand interactions highlighted BMSCs as key hub nodes in cellular networks. PPI network analysis revealed 46 nodes and 76 edges, with *GAPDH* as the top hub gene, followed by *MMP2*, *COL1A2*, *DCN*, *SPARC*, *COL5A2*, *COL5A1*, *TGFβ2*, *PRDX1*, and *TGFβI*, all of which were upregulated in BMSCs.

**Fig 6:**
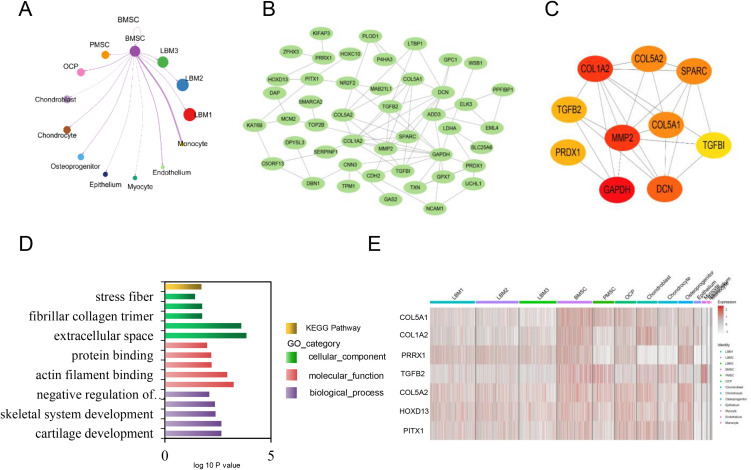
Analysis of differentially expressed genes (DEGs) in chicken BMSCs. **A.** Interaction network between BMSCs and other cells. **B.** Protein-protein interaction network (PPI) constructed from DEGs in BMSCs. **C.** Top 10 hub genes with higher degree of connectivity in BMSCs. **D.** Significantly enriched GO terms and KEGG pathways were identified for the DEGs in BMSCs. **E.** Heatmap of DEGs in BMSCs.

GO enrichment analysis emphasized key biological functions related to cartilage development and skeletal system development, with significant genes including *COL1A2*, *TGFβ2*, *COL5A2*, *PRRX1*, *DDRGK1*, *PITX1*, and *PRDX1*. t-SNE analysis confirmed high expression of *GAS2*, *COL5A1*, *COL1A2*, *PRRX1*, and *TGFβ2* in BMSCs. Intersection analysis pinpointed *COL5A1*, *COL1A2*, *PRRX1*, and *TGFβ2* as key marker genes, which were validated through immunofluorescence staining (Fig 6A-6D).

### Verification of marker gene expression in BMSCs with immunofluorescence technology

This study focused on identifying key biomarker genes in BMSCs within chicken limb buds. Through scRNA-seq analysis, we identified four significantly upregulated genes in BMSCs: *COL5A2*, *COL1A2*, *PRRX1*, and *TGFβ2*. To validate these findings, immunofluorescence staining was performed on limb bud sections from HH29-stage chicken embryos.

**Fig 7:**
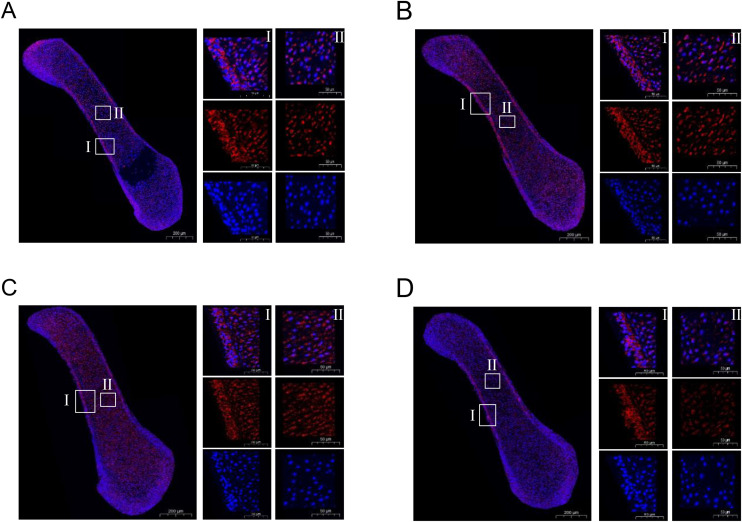
Immunofluorescence images showing the expression of COL5A2, COL1A2, PRRX1, and TGFβ2 in chicken hindlimb bud sections. Expression of COL5A2, COL1A2, PRRX1, and TGFβ2 was detected in the periosteal region and the primary ossification centers. **A.** Merged and single-channel images of COL5A2 (red) and DAPI (blue) staining. **B.** Merged and single-channel images of COL1A2 (red) and DAPI (blue) staining. **C.** Merged and single-channel images of PRRX1 (red) and DAPI (blue) staining. **D.** Merged and single-channel images of TGFβ2 (red) and DAPI (blue) staining. All experiments were independently repeated at least three times. Scale bars in snapshot image, 200 μm; scale bars in magnified images, 50 μm.

Immunofluorescence analysis demonstrated that *COL5A2*, *COL1A2*, *PRRX1*, and *TGFβ2* were predominantly localized within the periosteal region of the limb bud. Additionally, a minor presence of these proteins was observed in the primary ossification centers, indicating their potential association with the invasion of skeletal progenitor cells into the cartilage template (Fig 7). Notably, *COL5A2* showed distinct localization on the articular surface of fetal bones with comparatively elevated expression levels.

## Discussion

During embryonic limb development, limb buds, formed from the mesoderm-derived mesenchyme and ectoderm-derived epidermis, undergo continuous growth to develop into four limbs, involving complex cellular compositions and molecular regulations [27]. scRNA-seq is a powerful tool for elucidating the regulation of skeletal progenitor cells and cell-type differentiation through cross-species analysis, identifying marker genes, and revealing conserved gene expression patterns that are essential for biological processes.

Cross-species studies have demonstrated high conservation of muscle and neuronal cells, with stromal and muscle cells sharing a common ancestral state. Functional similarities between secretory and neuronal cells in vertebrates indicate convergent evolution [28]. In scRNA-seq analyses of the intestinal epithelium across various species, key cell types are conserved, although the *CA7*^*+*^ cell type is unique to humans, monkeys, and pigs [29]. Similarly, gene expression patterns in kidney macrophages and amygdalar neurons are highly conserved across species, including birds and mammals [30,31].

Feregrino et al. [17] used scRNA-seq to classify 10 cell types in HH29-stage chicken embryos, mainly skin, stromal, and blood cells. Our study revealed a significant overlap in cell composition between HH29-stage chicken embryos and human limb buds at 5 and 8 WPC, suggesting a developmental correlation at approximately 21 days of human gestation. Further studies are required to refine this timeline. He et al. [9] identified macrophages in human limb buds via scRNA-seq, originating from mononuclear precursors, and updated marker-gene comparisons, suggesting that these correspond to mononuclear cells.

Cross-species functional enrichment analysis revealed conserved gene functions. Li et al. [29] found that genes related to structure, size, amino acid transport, and metabolism were conserved across species. Similarly, the age-related downregulation of genes in mice, zebrafish, and other species has been linked to ATP metabolism and oxidative phosphorylation [32]. GO enrichment of DEGs also supports cell classification, with analysis confirming five major immune cell types in peripheral blood mononuclear cells across 12 species [33].

Our study found that gene functions in chicken and human LBM1–3, PMSC, BMSC, and OCP cells were highly conserved. GO enrichment analysis helped define the cell types in embryonic limb buds, with 13 cell clusters identified in chicken (HH29) and 16 in human (5WPC and 8WPC) limb buds. Consistent marker gene expression across clusters indicated that these markers reflected the characteristics of their respective cell groups. Pseudotime trajectory analysis showed BMSCs arise from early-stage mesenchymal progenitor cells (LBM1–3) but have not fully differentiated.

GO enrichment of chicken BMSCs highlighted their roles in extracellular matrix binding, cartilage development, and skeletal system pathways. KEGG analysis emphasized the TGF-β signaling pathway, which regulates mesenchymal stem cell proliferation and differentiation during embryonic development. Members of the TGF-β family, including bone morphogenetic proteins (BMPs), are key regulators in stem cell differentiation, playing a critical role in limb bud development [34–36].

Our findings showed that BMSCs expressed high levels of PDGFRA, CXCL12, and CD200, similar to DEGs in adult mSSCs, suggesting that BMSCs are part of a broader SSC population, likely originating from skeletal tissue progenitors [9,37]. Through PPI, GO, KEGG, and t-SNE analyses, we identified four key candidate genes—*COL5A2*, *COL1A2*, *PRRX1*, and *TGFβ2*—as potential BMSC biomarkers in chicken limb buds. PRRX1 is known for its role in self-renewal and pluripotency and is a recognized marker of periosteal-derived SSCs [24,38]. COL5A2 and COL1A2 encode critical collagen proteins involved in skeletal development, where their deficiencies can result in disorders [39–42]. TGFβ2 promotes BMSC proliferation and differentiation, vital for bone development and repair [43,44].

In this study, we identified 104 DEGs in chicken BMSCs, 31 of which were homologous to human BMSC genes, including *COL5A1*, *COL5A2*, *COL1A2*, *PITX1*, and *CD99*. This highlights both conserved and species-specific BMSC gene expression patterns in chickens and humans. Although these genes are not widely recognized as human BMSC markers, the limited research on SSC markers suggests that many important genes remain unexplored. Our findings provide insights into the identification of chicken BMSCs and contribute to a broader understanding of SSC biology.

Immunofluorescence is widely used to validate novel feature genes in scRNA-seq studies [45]. In human BMSCs, *CADM1* and the transcription factors *FOXP1/2* are expressed specifically in SSCs [9,46]. In mice, *CD168* BMSCs were enriched in the limb bud mesenchyme at E11.5, localized in the primary ossification center at E15.5, and observed on the fetal bone articular surface at E18.5, with *COL2A1* validated in similar regions [24]. In this study, *COL5A2*, *COL1A2*, *PRRX1*, and *TGFβ2* in chicken limb bud BMSCs were localized primarily in the periosteal region, with minor expression in ossification centers. These findings establish these genes as markers of chicken SSCs and suggest earlier skeletal development in chickens than in mice. These findings provide valuable insights into identifying marker genes for chicken SSCs and a foundation for understanding the unique aspects of poultry skeletal development.

Moreover, the four key candidate genes identified—COL5A2, COL1A2, PRRX1, and TGFβ2—likely play complementary roles in regulating skeletal stem cell behavior during limb development. COL5A2 and COL1A2 contribute to the structural integrity of the extracellular matrix, providing scaffolds for SSC proliferation and differentiation. PRRX1, a transcription factor, may govern SSC self-renewal and lineage specification, whereas TGFβ2 signaling modulates proliferation and differentiation of SSCs into osteogenic and chondrogenic lineages. Together, these genes may orchestrate a coordinated program that ensures proper bone and cartilage formation in the developing limbs. Their conserved expression in chicken and human BMSCs highlights their potential as universal markers and functional regulators of vertebrate skeletal development.

One limitation of this study is the small sample size and sequencing depth of chicken embryonic limb buds. However, further in vivo and in vitro experiments are required to confirm the presence of SSCs. The SSC marker genes identified require further investigation to clarify their biological functions. Understanding the roles of these genes in bone development, maintenance, and repair may facilitate the development of stem cell-based therapies for bone-related diseases.

## Conclusions

In conclusion, this study revealed strong conservation of cell types and functions between chicken and human embryonic limb buds, with notable similarities in the gene expression patterns of chicken SSCs and their human counterparts, despite some heterogeneity. *COL5A2*, *COL1A2*, *PRRX1*, and *TGFβ2* were identified as potential marker genes for chicken SSCs. Future studies should account for this heterogeneity and use appropriate markers and methodologies to enable precise classification and isolation. Although further validation is needed, this study provides a foundational framework for understanding the cross-species cellular composition and offers valuable insights for selecting animal models for skeletal biology research.

## Supporting information

S1 Fig Cross-species cell composition within integrated clusters. A. Proportions of HB and CB cells within each integrated cluster for the HH25 chicken hindlimb dataset. B. Proportions of HB and CB cells within each integrated cluster for the HH29 chicken hindlimb dataset. C. Proportions of HB and CB cells within each integrated cluster for the HH31 chicken hindlimb dataset.
(PDF)

S1 Table Differentially expressed genes in all cell types of human and chicken limb buds. (XLSX)
