# Supplementary figures and images for: Single-cell profiling unveils key regulators of skeletal stem cells in chicken and human embryonic limb development
Source: PLoS One. 2026 Apr 28;21(4):e0346514. doi: 10.1371/journal.pone.0346514 (PMC13123952; doi:10.1371/journal.pone.0346514)

A

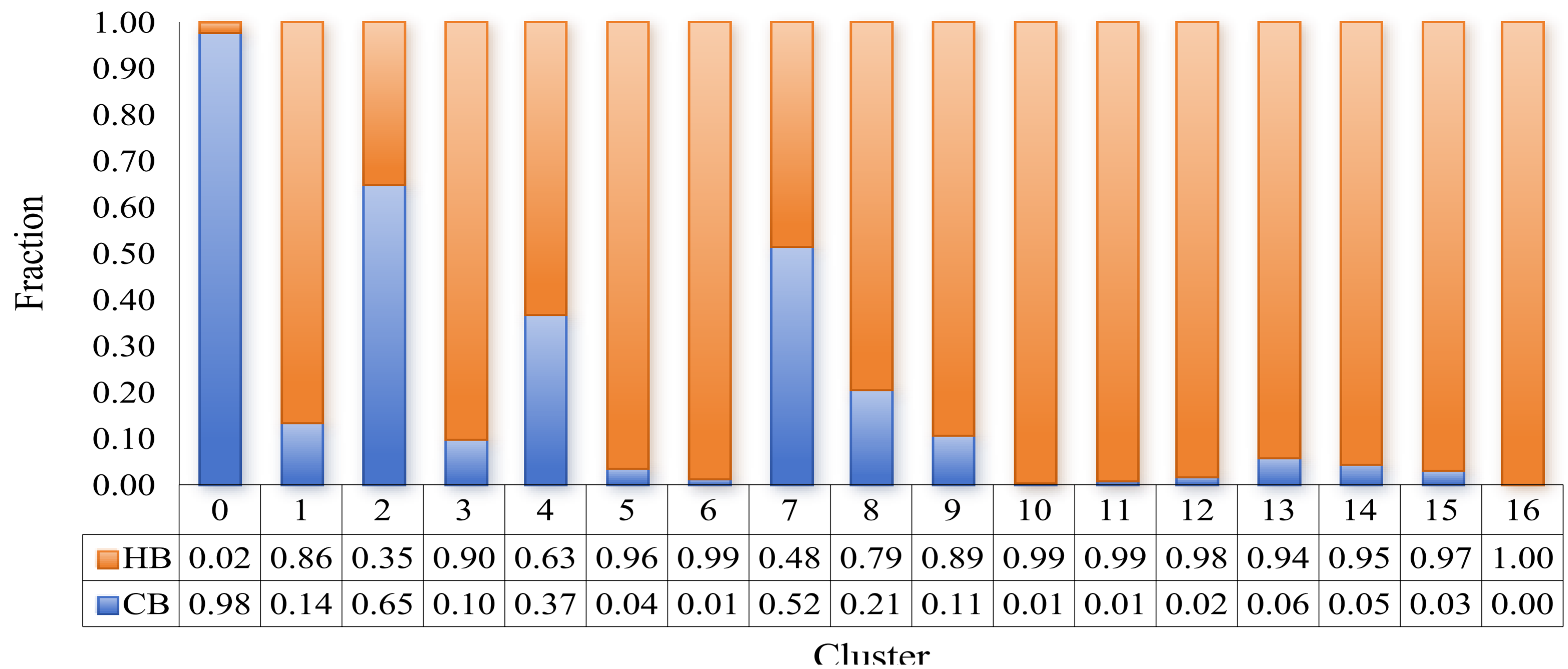

B

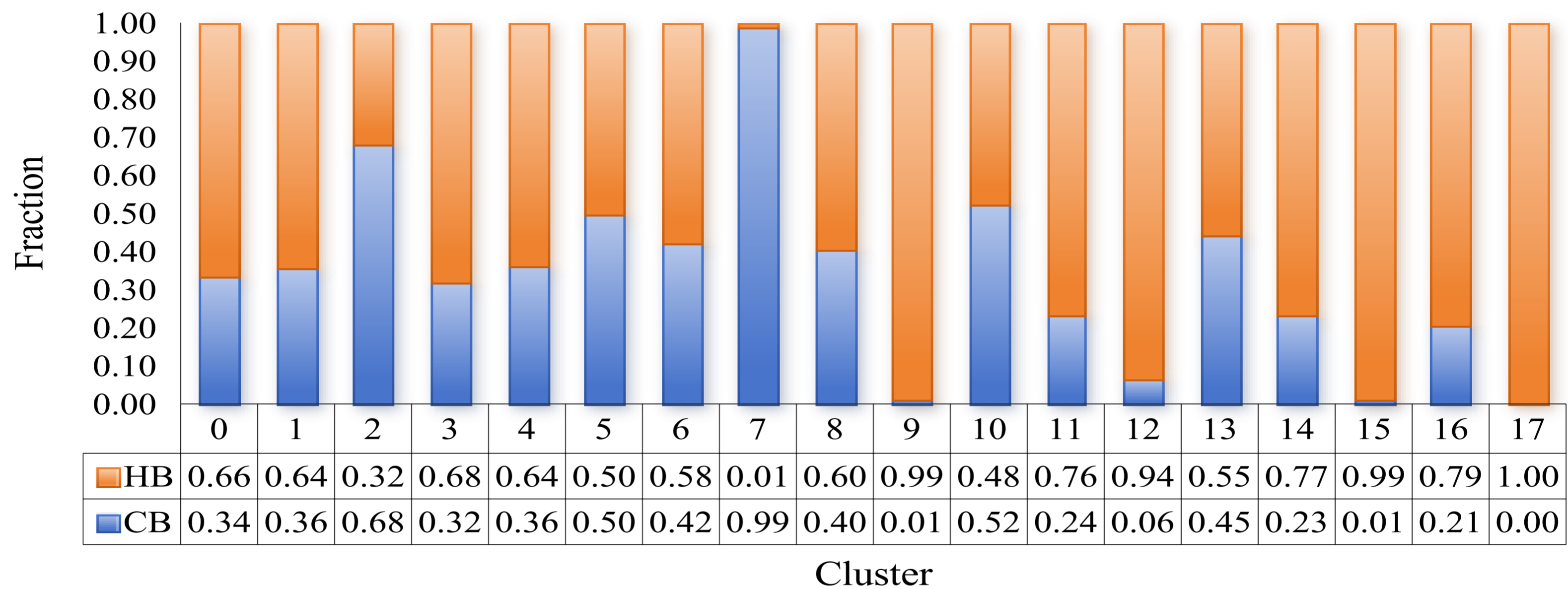

C

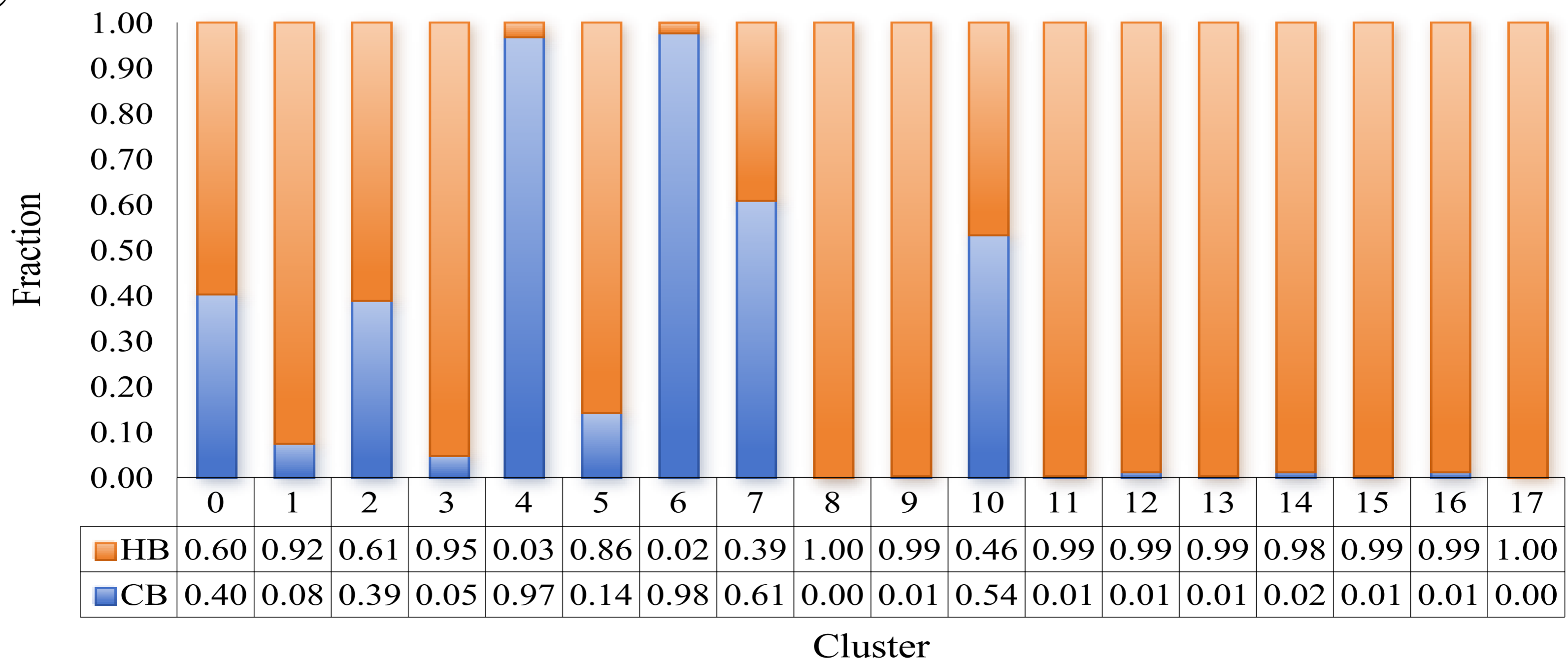

Supplement: S1 Fig — A. Proportions of HB and CB cells within each integrated cluster for the HH25 chicken hindlimb dataset. B. Proportions of HB and CB cells within each integrated cluster for the HH29 chicken hindlimb dataset. C. Proportions of HB and CB cells within each integrated cluster for the HH31 chicken hindlimb dataset. (PDF) [file pone.0346514.s001.pdf]
